# Implementing educational interventions and key performance measures sustains quality of endoscopic assessment in patients with Barrett’s esophagus

**DOI:** 10.1055/a-2542-0618

**Published:** 2025-03-14

**Authors:** Deloshaan Subhaharan, Pradeep Kakkadasam Ramaswamy, Mark Jones, Sneha John

**Affiliations:** 1 60093 Department of Digestive Health, Gold Coast University Hospital, Gold Coast, Australia; 2 3555 Health Sciences and Medicine, Bond University Ltd, Gold Coast, Australia

**Keywords:** Endoscopy Upper GI Tract, Barrett's and adenocarcinoma, Quality and logistical aspects, Training, Quality management

## Abstract

**Background and study aims:**

Quality metrics for Barrett’s esophagus (BE) are anticipated to improve outcomes for patients through earlier detection of neoplasia. The European Society of Gastrointestinal Endoscopy has developed guidelines to homogenize endoscopic quality in BE. Our study aimed to assess the impact of recommended key performance measures (KPMs) and their sustainability.

**Patients and methods:**

A single-center, retrospective study (Phase 1) was conducted over 8 weeks. The KPMs assessed were: 1) pre-procedure metrics including indication, consent, safety checklist (target of 100%); and 2) Prague classification, Seattle protocol, or targeted biopsies, inspection time of 1 minute per cm, advanced imaging and surveillance recommendations (target of 90%). Following baseline analysis, multimodal educational interventions were implemented and repeated at 6-month intervals. Repeat analysis was performed at 6 months and 1 and 3 years (Phases 2, 3 and 4 respectively).

**Results:**

In Phase 1, 39 patients with BE underwent endoscopy. Phase 2 evaluated 40 patients with BE. Phase 3 analyzed 59 patients with BE, and Phase 4 identified 34 patients with BE. Pre-procedure metrics were met in 100% of patients across the 3-year period. Baseline analysis displayed suboptimal performance at 45% to 75% for all other KPMs. However, after regular multimodal educational interventions, quality standards significantly improved and were able to be maintained over all phases, achieving pre-set targets of >9 0% for all KPMs except one.

**Conclusions:**

Sustaining improvements in quality metrics in Barrett’s endoscopy is important. Our study suggests that regular, replicable education interventions have a positive effect and allow sustained long-term improvements in quality metrics.

## Introduction

Barrett’s esophagus (BE) is a premalignant condition in which normal squamous mucosa is transformed to metaplastic columnar epithelium 1 . Risk factors for BE include gastroesophageal reflux disease, family history, male gender, obesity, and smoking 2 . It has a well-established linear progression from non-dysplastic epithelium to dysplasia, and subsequently to adenocarcinoma 3 . Incidence of BE, and subsequently esophageal adenocarcinoma, has rapidly risen over the last few decades in developed countries 4 . Early detection, management, and appropriate surveillance of BE thus is crucial to prevent progression in individuals at risk.

BE length is defined as long segment (≥ 3 cm) or short segment (< 3 cm). Current consensus recommends endoscopic surveillance for BE to detect and treat early neoplastic lesions to prevent development of adenocarcinoma 5 . Those detected with dysplasia are typically referred for endoscopic treatment with a success rate of 85%, reducing the need for surgical management, which is associated with higher morbidity and mortality 6 .

Recent technological advances have allowed clinicians to improve BE assessment, and hence, earlier detection of dysplasia. Quality assurance, therefore, is important to improve efficacy and ensure high-quality care that correlates with patient outcomes. The European Society of Gastrointestinal Endoscopy (ESGE) has developed consensus guidelines in order to homogenize quality in endoscopic assessment of BE 7 . The fundamental recommendations include use of Prague classification, advanced imaging, Seattle protocol or targeted biopsies, adequate examination time, and appropriate surveillance recommendations. In routine clinical practice, adherence to these guidelines is variable among clinicians, which may impact on dysplasia detection and hence patient outcomes 8 . This is likely more pronounced in non-academic centers.

Regular educational interventions have been demonstrated to improve overall quality of colonoscopy and patient outcomes 9 . Although previous studies, including a recent video-based assessment, have evaluated adherence and quality in BE, there are currently no studies that assess this long-term 10 . The primary aim of our study was to assess compliance with current ESGE guidelines and evaluate whether regular educational interventions sustain quality improvement long-term 7 . Secondary outcomes assessed were whether there was an increased dysplasia detection rate (DDR) by complying with these quality standards.

## Patients and methods

### Study design

This was a single-center retrospective cohort study from a tertiary academic center. The study was approved by the Gold Coast Health Service Human Research Ethics Committee (Ref: LNR/2019/QGC/53563).

**Fig. 1:**
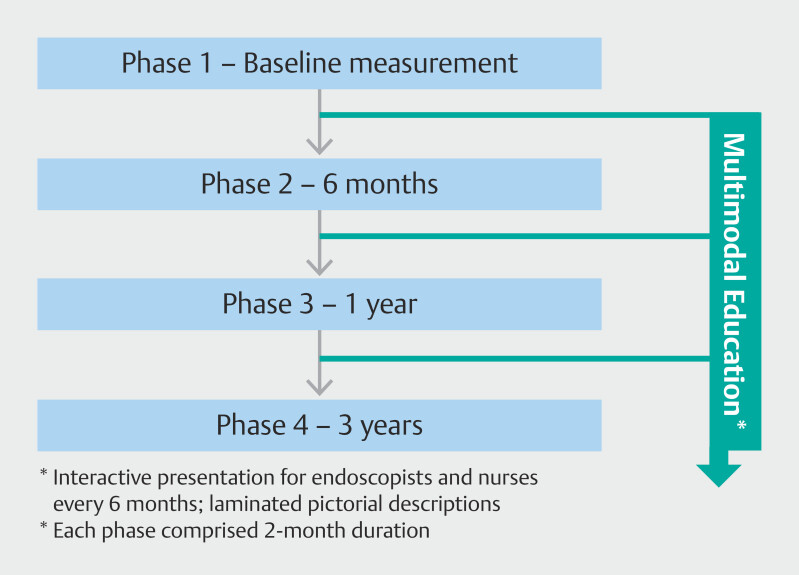
Schematic of study design.

The study was divided into four distinct phases over a 3-year timeframe ( Fig. 1 ). Phase 1 comprised a retrospective audit of current practice over an 8-week period from March 1 to April 30, 2019 to establish baseline quality practice against the established key performance measures (KPMs). Multimodal educational interventions were commenced with the first session in July 2019 for endoscopists and endoscopy nurses with 6-monthly multimodal review sessions thereafter. Phase 2 was conducted between October 1 to November 30, 2019. Similarly, Phases 3 and 4 were performed over similar time frames between September 1 and October 31, 2020 and between June 1 and July 31, 2022.

### Study population

All consecutive adult patients (≥ 18 years of age) who underwent diagnostic gastroscopy for confirmed BE were included in the analysis. Clinical, histopathological, and endoscopic reports were collected from electronic medical records. Patients were excluded if there was presence of underlying varices, severe esophagitis, or if the procedure was intended solely for the purpose of therapeutic intervention of previously detected neoplasia. All procedures were performed with anesthetist-administered sedation and a standard diagnostic gastroscope with high-definition white-light and virtual chromoendoscopy imaging modalities. Endoscopy software (ProvationMD v7.15) was utilized to create endoscopic reports and images that allow for both free-text and/or automated prompt format. All endoscopists (gastroenterologists including experts and non-experts) were included in quality analysis after obtaining informed consent. Experts were defined as those with > 5 years of clinical endoscopic experience and/or advanced endoscopic imaging and therapeutic skills. The study included 30 proceduralists of whom 50% were experts and 50% were non-experts.

### Multimodal education

**Fig. 2:**
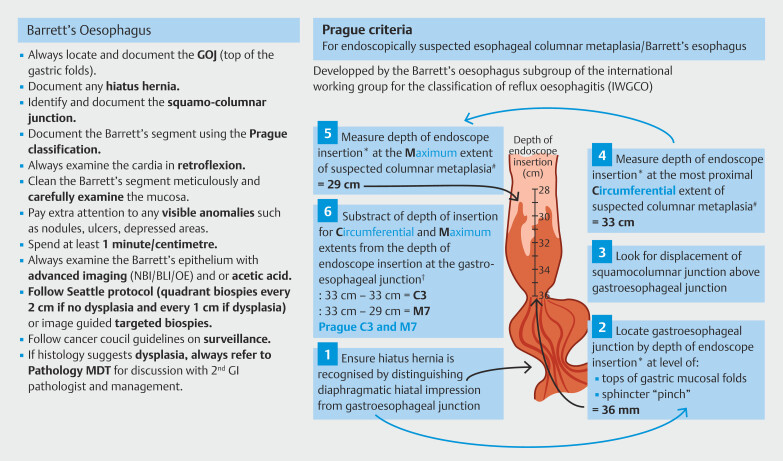
Pictorial description of quality standards displayed in endoscopy suites.

BE educational intervention was delivered in a multimodal fashion using up-to-date published guidelines 7 . All educational material was electronically distributed to all endoscopists and nurses. These included:

1. Interactive in-person presentation of approximately 1-hour duration highlighting recommendations and guidelines through high-definition images and videos. The presentation emphasized accurate identification of anatomical landmarks (gastroesophageal junction and squamocolumnar junction), measurement of maximal BE extent, Prague classification, Seattle protocol, surveillance recommendations, and importance of advanced imaging techniques to improve recognition of visible lesions. The index session was delivered on July 22, 2019. This was repeated every 6 months thereafter. The department has an established quality and safety program, which provides individual feedback to low-performing endoscopists in both colonoscopy and gastroscopy. This was utilized after analysis of each retrospective cohort. Targeted feedback was not provided otherwise to endoscopists.
2. Endoscopy nursing education was performed as part of the monthly endoscopy unit meetings coordinated by a clinical nurse facilitator. High-definition images and videos along with descriptions of adequate biopsy protocols, accurate labelling of specimens, and use of acetic acid were the key components.
3. Laminated pictorial descriptions for all BE quality standards, provided as visual cues in each endoscopy suite ( Fig. 2 ), were available throughout the entirety of the study. They were easily visible and accessible to both medical and nursing staff.

### Quality metrics and targets

**Fig. 3:**
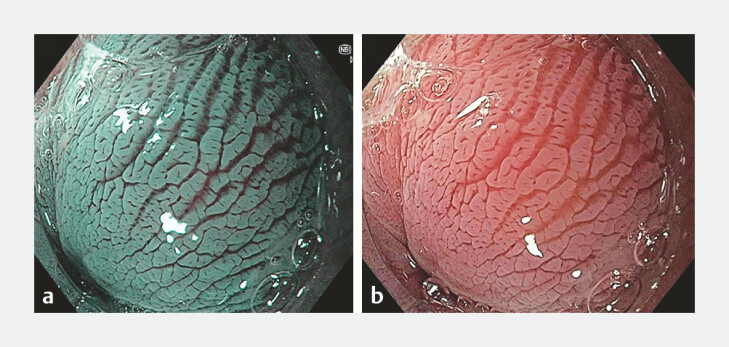
**a** Narrow-band imaging and **b** acetic acid with near-focus.

Endoscopist compliance with pre-procedure metrics and KPMs were evaluated. Pre-procedure metrics included appropriate indication, consent, and safety checklist with a target of 100%. All other KPMs had a target of 90%. They included:

1. Use of Prague classification. This is a validated classification system used in order to standardize reporting of BE by detailing the location of the top of the gastric folds, the proximal extent of the circumferential (C) BE segment, and the maximal (M) extent 11 .
2. Inspection time of at least 1 minute per centimeter. This has been shown to be significantly associated with improved detection of early lesions 12 13 . This was aided by the endoscopy software ProvationMD, whereby endoscopy nursing staff are manually able to click “Start Withdrawal” and “Pause” to obtain an accurate withdrawal time instead of approximating with total start and end time.
3. Use of advanced imaging and/or acetic acid. This can enhance subtle mucosal and vascular abnormalities that may indicate presence of early neoplasia and has been shown to improve diagnostic yield of targeted biopsies by 15 times compared with random biopsies 14 . The study mainly used narrow band imaging (NBI) and near-focus for advanced imaging with a small number using blue light imaging ( Fig. 3 **a** and Fig. 3 **b** ).
4. Use of Seattle protocol or image-guided targeted biopsies. As per the Seattle protocol, quadratic biopsies every 2 cm of BE length are required unless there is suspected or known dysplasia, whereby biopsies are taken every 1 cm. If visible mucosal abnormalities are detected, targeted biopsies should be obtained. This has been shown to increase detection of advanced lesions 15 16 .
5. Surveillance recommendation against our current national cancer council guidelines 17 . The aim of surveillance is to detect dysplasia and early cancer for early treatment. Frequency of surveillance is based on the length of BE and presence or absence of dysplasia.

### Outcomes

The primary outcome was to assess compliance with current BE guidelines; co-primary outcome was to evaluate whether regular educational interventions can sustain quality improvement long-term. The secondary outcome assessed was whether there was an increased DDR by complying with these quality standards when compared with the baseline. This was calculated as the proportion of patients with confirmed dysplasia (indefinite [IND], low-grade dysplasia [LGD] or high-grade dysplasia [HGD]) or adenocarcinoma (EAC) against total BE cases. All cases of dysplasia were reviewed by two expert gastrointestinal pathologists and reviewed in a multidisciplinary pathology meeting.

### Statistical analysis

Results were reported as number of BE cases detected and percentage of cases that complied with the performance measures. For comparison with the percentage of cases that complied with the performance measures from baseline to follow up, Fisher’s exact or Chi-square tests were used. A two-tailed *P* < 0.05 was considered statistically significant. All analysis was performed using Stata 15 (StataCorp LLC, College Station, Texas).

## Results

**Fig. 4:**
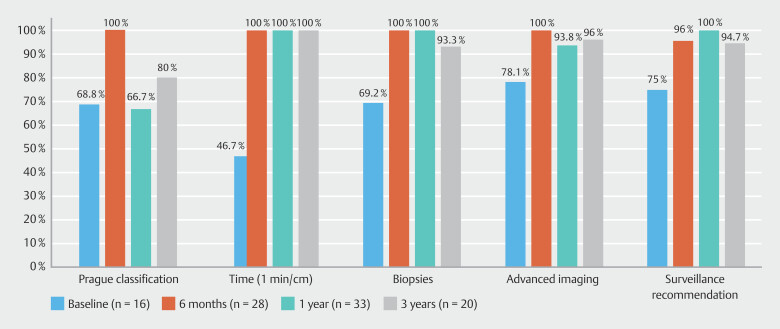
Quality performance in long-segment Barrett’s esophagus.

**Fig. 5:**
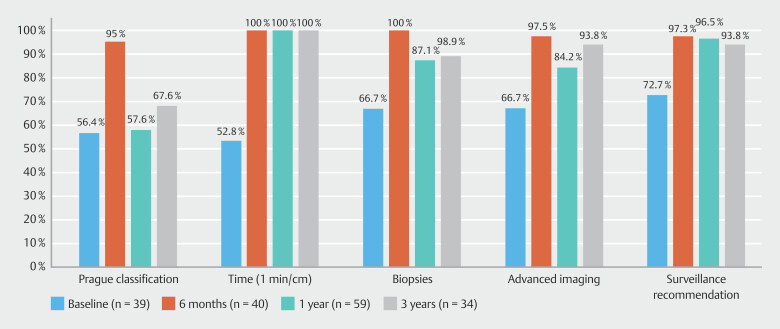
Quality performance in total Barrett’s esophagus.

**Fig. 6:**
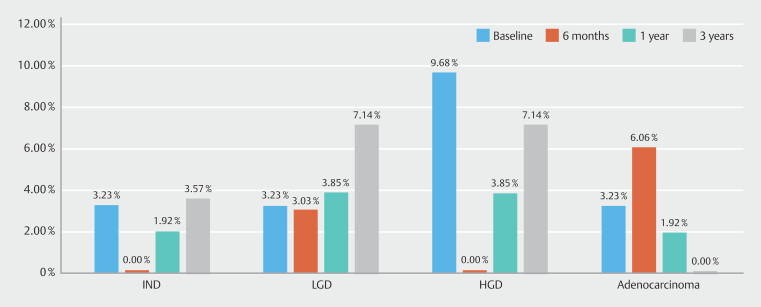
Dysplasia detection rate.

**Fig. 7:**
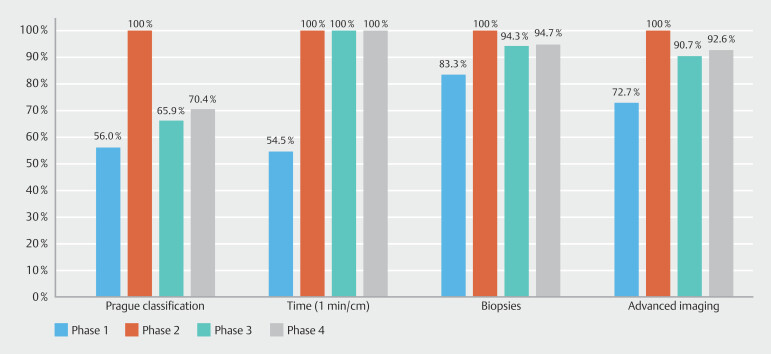
Quality performance among expert endoscopists.

**Fig. 8:**
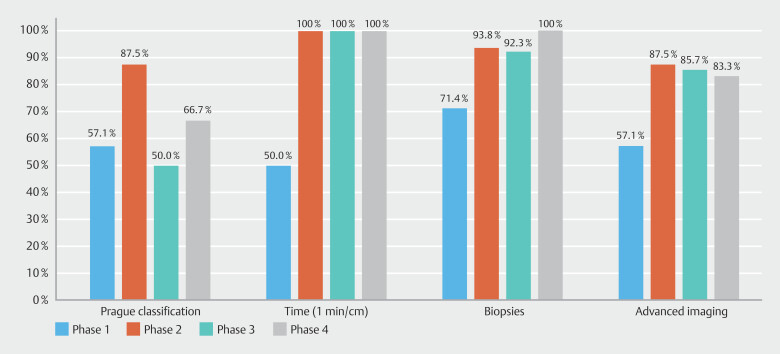
Quality performance among non-expert endoscopists.

Over the study period, 3396 diagnostic gastroscopies were performed: 804 in Phase 1, 823 in Phase 2, 867 in Phase 3, and 902 in Phase 4. Rates of adherence to each KPM for long-segment and total BE are shown in Fig. 4 and Fig. 5 , respectively. Total dysplasia rates are shown in Fig. 6 . Further subgroup analysis comparing performance of expert and non-experts is shown in Fig. 7 and Fig. 8 , respectively. 100% of pre-procedure metrics were obtained and this was maintained throughout the entirety of the study. All endoscopists participated in at least one interactive educational session and all had access to visual reminders of quality in the endoscopy suite for every procedure.

### Phase 1

There were a total of 39 cases of BE identified by 14 endoscopists (8 experts), with 16 (41%) being long-segment BE. Phase 1 displayed suboptimal performance for both total and long-segment BE as per ESGE targets for each KPM. This was most substantial for inspection time in long-segment BE at 46.7%, followed by use of Prague classification in 68.8%, adequate biopsies in 69.2%, adequate surveillance recommendation in 75%, and use of advanced imaging in 78.1%. Similar results were seen for total BE. In all cases, targeted biopsies were performed in 46.9% and random biopsies in 31.3%. In regard to dysplasia, there was one case of LGD and three HGDs and one IND were identified, with overall DDR of 12.8%. Visible lesions were seen in 80% of dysplasia cases.

### Phase 2

There were a total of 40 cases of BE identified by 16 endoscopists (9 experts), with 28 (70%) being long-segment disease. Phase 2 showed a significant improvement for each KPM in both long-segment and total BE achieving targets of above 90%. For long-segment BE, 100% was achieved for Prague classification ( *P* = 0.002), inspection time ( *P* < 0.0001), adequate biopsies ( *P* = 0.002), and use of advanced imaging ( *P* = 0.006), with 96% for surveillance recommendation ( *P* = 0.03). In all cases, targeted biopsies were performed in 42.4% and random biopsies in 54.5%. There were one LGD and two EACs identified with DDR 7.5%. Visible lesions were seen in 66.7% of dysplasia cases.

### Phase 3

There were a total of 59 cases of BE identified by 18 endoscopists (12 experts), with 33 (56%) being long-segment disease. For long-segment BE, KPMs were able to be sustained above target of 90% with the exception of Prague classification at 66.7% ( *P* = 0.88). For total BE, KPM targets were partially achieved with 57.6% for Prague classification ( *P* = 0.91), 100% inspection time ( *P* < 0.0001), 87.1% adequate biopsies ( *P* = 0.02), 84.2% use of advanced imaging ( *P* = 0.04), and 96% with adequate surveillance recommendation ( *P* < 0.0001). In all cases, targeted biopsies were performed in 52.1% and random biopsies in 41.7%. There were two LGDs, two HGDs, and two EACs identified with DDR 10.2%. Visible lesions were seen in 66.7% of dysplasia cases.

### Phase 4

There were a total of 34 cases of BE identified by 17 endoscopists (8 experts), with 20 (59%) being long-segment disease. Phase 4 showed that quality targets above 90% were able to be largely preserved with the exception of Prague classification in 80% and 67.6% for long-segment and total BE, respectively. In long-segment BE, targets of 100% for inspection time ( *P* < 0.0001), 93.3% adequate biopsies ( *P* = 0.04), 96% use of advanced imaging ( *P* = 0.08), and 94.7% surveillance recommendation ( *P* = 0.08) were achieved. In all cases, targeted biopsies were performed in 72% and random biopsies in 24%. There were two LGDs and two HGDs identified with DDR 11.8%. Visible lesions were only seen in 25% of dysplasia cases.

## Discussion

With rising incidence of BE and EAC worldwide along with rapid advance in endoscopic therapy, there is a growing need to detect dysplastic changes as early as possible. While only a small cohort of patients with BE go on to develop EAC, advanced neoplasia has a very high impact on patients and costs to the healthcare system. Therefore, high-quality screening and surveillance for BE and minimally invasive endoscopic management is essential to achieve good patient outcomes and cost-effective care.

Previous studies have shown poor compliance with quality metrics and clinical guidelines with adherence rates of only up to 50% for Prague classification and Seattle protocol 8 10 18 19 20 . Not unexpectedly, the initial phase of our study also displayed suboptimal performance with respect to KPMs. More recent studies have shown that educational interventions improve adherence to quality standards. However, they have not shown that quality improvements can be sustained long-term. It has been shown that multimodal education interventions enhance quality of upper gastrointestinal endoscopy for premalignant diseases by improving compliance with current guidelines and increasing detection of clinically significant pathology 21 . Other studies have shown quality improvements per the American Society for Gastrointestinal Endoscopy guidelines, along with formal training in BE significantly improving DDR 22 23 . The AQUIRE study was a multicenter, randomized, controlled trial in the United States in which gastroenterologists were assigned to receive a structured intensive education program over 6 months and were compared with local standard practice 24 . They showed that Seattle protocol biopsies significantly improved in the intervention arm; however, they did not look at other KPMs.

Our study is the first to demonstrate that regular multimodal educational interventions significantly improve overall quality of endoscopic assessment in BE and crucially, these can be sustained. The simple nature of the educational modalities makes them replicable and they can be utilized in academic and non-academic hospitals. In particular, Seattle protocol biopsies, inspection time, use of advanced imaging, and appropriate surveillance recommendations improved the most. This is most likely due to use of pictorial descriptions in each endoscopy suite along with the unique impact of nursing education, which served as more constant reminders of the quality standards required. Nursing education allowed procedure staff to play a more active role by monitoring inspection time and reminding endoscopists about use of advanced imaging and biopsy protocols if not adhered to. We believe that this is particularly pertinent for non-expert endoscopists and those who newly begin work in our department and may be unfamiliar with the published standards. Over the 3-year time-frame, utilization of Prague classification was the quality metric that was least adhered to in our study. It was noted that all proceduralists described the length of the BE segment in detail on their report but did not always utilize the Prague classification for this purpose. The prompt for Prague classification in our reporting software was not as readily visualized or automated compared with the other quality markers. This technical issue, therefore, may be the cause for some attrition over time, rather than a true loss of adherence to this quality standard.

Quality standards for use of advanced imaging, Seattle protocol biopsies, and surveillance recommendations were numerically higher in patients with long-segment BE when compared with those with short-segment BE. Initial multimodal education post-Phase 1 showed a significant improvement across each KPM for both long-segment and total BE. After regular education interventions, quality standards were able to be largely maintained over a 3-year period, achieving target of above 90%. Use of advanced imaging particularly in long-segment BE is crucial to detect subtle lesions. After Phase 1, whereby adherence was only 78.1%, subsequent phases showed adherence rates of 100%, 93.8%, and 96%, respectively. Among expert endoscopists ( Fig. 7 ), initial analysis highlighted that they were not adherent to the recommended guidelines; however, after multimodal education, the expert endoscopists sustained quality throughout the study (except for Prague classification). Of note, the non-expert endoscopists significantly improved on most quality standards, which underscores the importance of multimodal education for all levels of endoscopists ( Fig. 8 ). The DDR between each phase did not significantly change, which is likely multifactorial given the low prevalence rate in Australia for BE-related dysplasia along with small numbers among each phase. Similarly, the true impact of sustaining quality markers on DDR and other clinical outcomes may require longer follow-up times with analysis for our entire annual cohort of patients, which we are currently in the process of performing.

There are several strengths with our study. First, a long follow-up period of 3 years with regular assessments and interventions has not previously been performed. This enables assessment of sustainable improvements across all KPMs in quality of BE endoscopy. Second, the study shows that quality metrics can be implemented and improved upon, across varying levels of experience including experts and non-experts. This suggests that multimodal education is imperative for all levels of experience to maintain quality standards. Third, this is the first study to involve nursing education as a component of improving quality standards in BE endoscopy.

There were a few limitations to the study. This was a single-center study and there was a lack of randomization (education vs. no education). The endoscopists were aware the study was being conducted after baseline collection of data only and did not have access to real-time data. This may have contributed to some bias for Phase 2. However, overall quality sustained improvement across all phases across different time periods unbeknown to the endoscopists, suggesting that the risk of bias is low. There were also a small portion of patients (6.98%) that were the same across the study period, which may have constituted some bias because the endoscopist had access to the previous report; however, at least 60% of these cases were from Phase 1 wherein quality underperformed. Across all the phases, there were slight differences in the number of endoscopists present, which may impact assessment of sustainability in certain endoscopists. However, only one-quarter were not present during the entirety of the study across all four phases, but all endoscopists were present for at least two phases. Finally, it was difficult to appreciate whether multimodal education interventions ultimately improved the DDR, given the low recorded prevalence of BE in the Australian population and lack of longer-term follow-up.

## Conclusions

In conclusion, this study underscores the positive impact of regular multimodal educational interventions for improving endoscopic assessment in BE in order to sustain current quality standards. The multimodal education strategies utilized were simple and easily replicable, and hence, can be implemented easily in both academic hospital and community-based settings. However, further studies are proposed to identify the best educational strategies to improve quality adherence globally. The role of artificial intelligence in this area is fast emerging and we look forward to further data.
